# A case report of an early gastrocolic fistula following Roux-en Y gastric bypass, a unique and uncommon complication

**DOI:** 10.1016/j.ijscr.2024.110191

**Published:** 2024-08-16

**Authors:** Mohammad Almayouf, Awadh Alqahtani

**Affiliations:** a Prince Sattam bin Abdulaziz University, College of Medicine, Department of Surgery, Alkharj, Saudi Arabia; b King Saud University, College of Medicine, Department of Surgery, Riyadh, Saudi Arabia

**Keywords:** Gastric bypass, Gastroplasty, Gastric fistula, Anastomotic leak, Stents, Bariatric surgery

## Abstract

**Introduction and importance:**

Gastrocolic fistula (GCF) following Roux-en-Y gastric bypass (RYGB) is uncommon. Usually it presents late with nonspecific symptoms and originates from the gastrojejunostomy (GJ). Management of such complication can be surgical, but endoscopic management can be implemented in selected patients. To our knowledge this is the first case reporting an early GCF originating from gastric pouch successfully managed with endoscopic stenting.

**Case presentation:**

A 54-year-old female, with surgical history of open vertical band gastroplasty (VBG), complaining of weight regain and reflux symptoms. The plan was to laparoscopically convert VBG to RYGB. Two weeks after, she presented unusually with only fatigue and epigastric pain.

**Clinical discussion:**

Leak was suspected and needed to be ruled out. The patient was presenting in an unusual presentation, i.e. vitally stable and only fatigued. Workup including laboratories, computed tomography, and endoscopy confirmed staple line disruption with development of early GCF. Management included endoscopic fully covered stent, total preantral nutrition.

**Conclusion:**

With a well-trained team and the availability of expertise, GCF can be managed with endoscopic stents.

## Highlights

• Gastrocolic fistula after Roux-en-Y gastric bypass in the context of bariatric surgery is fairly rare.
• Fistula forming at gastrojejunostomy are the most common, presenting as a late complication, with nonspecific symptoms.
• Our case is unique in its location, time interval, and how it was successfully managed non surgically.

## 1 Introduction

Until now, bariatric surgery (BS) has been the most effective option for the treatment of morbid obesity compared to other modalities. Hence, the number of primary BS conducted worldwide has increased exponentially [1,2]. Despite that, some patients might need to be re-operated for the possibility of weight regain or side effects of the primary BS. Revisional bariatric surgery has a noteworthy rate of postoperative complications because of higher technical demand, longer operative time, and operating on disturbed tissue [[3], [4], [5]].

Vertical banded gastroplasty (VBG) was introduced by Mason in the 1980s, which entails creating a small gastric pouch by vertically stabling the stomach from a created punch hole to the Angle of His and restricting the outlet with a band between the hole and the lesser curvature. With the development of more effective BS and the evident VBG failure (weight regain, gastroesophageal reflux (GERD)) the procedure became obsolete and more prone to conversion [6]. One option for converting VBG is the conversion to Roux-en-Y- gastric bypass (RYGB), and the anastomotic leak is a feared complication post-conversion. Because of the nature of the procedure and multiple constructed staple lines, leaks can potentially occur in different areas with varying degrees of presentation and diagnostic challenges. The pathophysiology of fistula formation can be attributed to malignancy, and with bariatric surgery, anastomotic disruption and reaction to foreign body (gastric band) are the most frequent causes [7]. Very few reports in the literature explained the management of such complication and all of the management reported were surgical. We present a case of an open VBG converted laparoscopically to RYGB complicated by an early gastrocolic fistula (GCF) located at the gastric pouch successfully managed with endoscopic stenting. As this is a case report, ethical approval was waived by the institutional review board at Dr. Sulaiman Alhabib Medical Group. Written informed consent was obtained from the patient for publication and any accompanying images. A copy of the written consent is available for review by the Editor-in-Chief of this journal on request. The work has been reported in line with the SCARE criteria [8].

## 2 Case report

### 2.1 Patient information

A 54-year-old female, with hypertension, heavy smoker (1–2 packs/day for 20 years), and body mass index of 46.54 kg/m^2^ presented to the obesity outpatient clinic complaining of weight regain, dysphagia, and reflux symptoms for four years. Twenty years ago, she underwent open VBG as a management for obesity, followed by open revision of the VBG one year after her index surgery because of dysphagia. The maximum weight she lost 85 kg. Since the surgery, she has had dysphagia to a solid and soft diet, GERD, and dependency on high doses of proton pump inhibitors (Dexlansoprazole 60 mg BID). Her surgical history was significant for open hernia repair and open total abdominal hysterectomy. On examination, her vitals were significant for elevated blood pressure, and the patient's weight/BMI were 126.7 kg/46.54 kg/m^2^ respectively. Abdomen was examined for any potential hernias. Preoperative workup (laboratories, cardiac workup, chest x-ray), upper gastrointestinal series (UGI) and esophagogastroduodenoscopy (EGD) were significant for a large divided pouch, gastritis, narrowing near the band, and stitches from the previous VBG. A multidisciplinary team counseling consisting of the bariatric surgeon, behavioral therapist, and dietician was made to ascertain the patient's goals and establish a plan. The patient was motivated and aimed to eliminate GERD and lose weight to improve her quality of life.

### 2.2 Operation and postoperative period

**Fig. 1:**
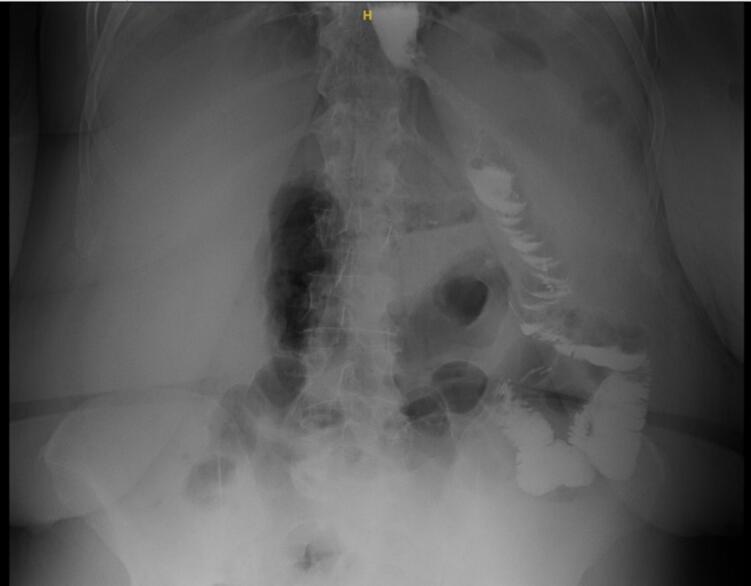
Post operative contrast study with no leak or contrast hold up.

Preoperative anticoagulation (5000 units unfractionated heparin) and antibiotics (2 g second-generation Cephalosporins) were administered, followed by general anesthesia induction and positioning. The surgery was conducted by a Fellow of the Royal College of Physicians and Surgeons of Canada trained surgeon, with over 1000 bariatric surgery cases annually. The Abdomen was accessed through Visiport™ 5 mm trocar at the left upper quadrant with no injury; then pneumoperitoneum was achieved with a set intrabdominal pressure of 15 mmHg. Other ports were inserted without issues, and a Nathasan retractor was used for liver retraction. The adhesions between the stomach and liver were released, dissection and delineation of the previous staple lines were accomplished, and there was no gastro-gastric fistula. The location of the previous mesh was noted. A window was created at a lesser curvature, and a horizontal stapler was applied away from the fibrous tissue; this was followed by vertical staplers under the guidance of a calibration tube. We consistently oversaw the newly created staple lines of the pouch. A gastrotomy was created, and then a gastrojejunostomy (GJ) was established with a linear stapler at 2.5 cm with a biliary limb of 120 cm; the GJ was closed with double layer hand-sewn technique using a 3–0 absorbable monofilament sutures. A side-to-side jejunojejunostomy (JJ) was established with 120 cm of alimentary limb and closed using double layer hand-sewn technique using 3–0 absorbable monofilament sutures. All newly established staple lines were oversewn, and hernia defects were closed with non-absorbable sutures. Termination of anesthesia and immediate postoperative period was unremarkable. Intravenous fluids, analgesics, anticoagulants, proton pump inhibitors, and antiemetics were instituted according to an established protocol. Vitals were measured regularly. A UGI showed no leak or contrasted hold-up the next day, reaching the colon (Fig. 1). The patient was discharged with clear instructions about her medications, dietary plan, and a two week follow-up appointment.

### 2.3 Follow up period

**Fig. 2:**
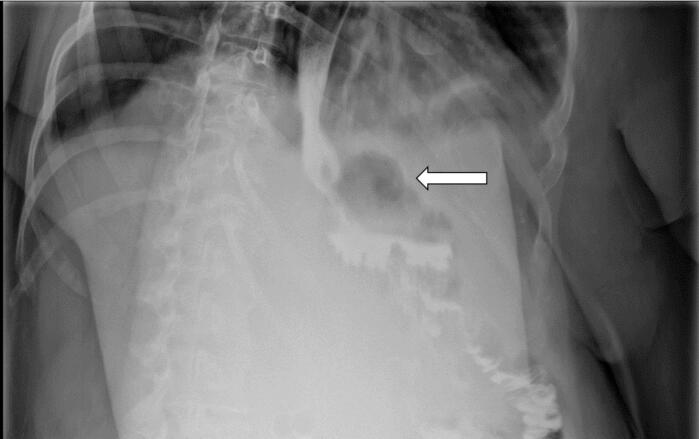
Two weeks post operative contrast study showing a cavity located just distal to GEJ.

**Fig. 3:**
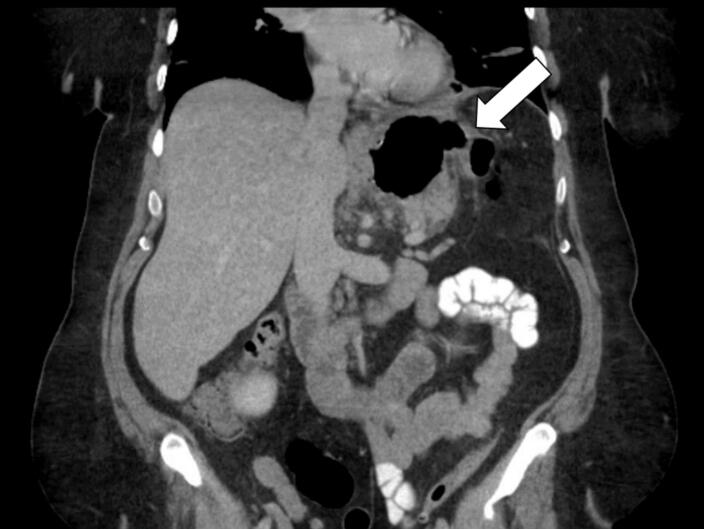
A computed tomography showing the cavity and an early gastrocolic fistula.

Two weeks after, the patient came to the clinic with complaints of severe nausea, mild epigastric pain, and fatigue. On examination, her vitals were unremarkable, and she looked dehydrated. She was admitted, kept NPO, rehydrated with intravenous fluids, and given multimodal IV antiemetics, proton pump inhibitors, and multimodal analgesics. After inquiry, the patient was consuming cigarettes and not drinking enough fluids. Laboratories were significant for mild leukocytosis of 12.9, and C-reactive protein of 250. A UGI showed a cavity filled with contrast just distal to the gastroesophageal junction, which was not present in a postoperative UGI (Fig. 2). Computed tomography with oral and IV contrast confirmed the presence of the cavity with a GCF (Fig. 3). The plan was to keep the patient NPO and start total parental nutrition, intravenous fluids, and broad-spectrum antibiotics.

**Fig. 4:**
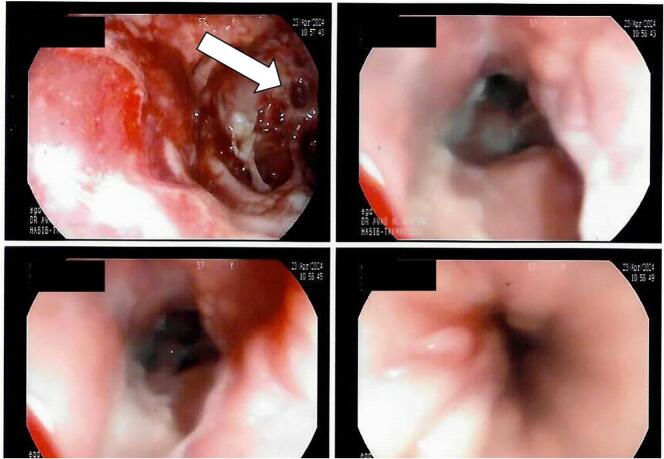
The gastroscopy showing the hyperemic cavity with the GCF.

**Fig. 5:**
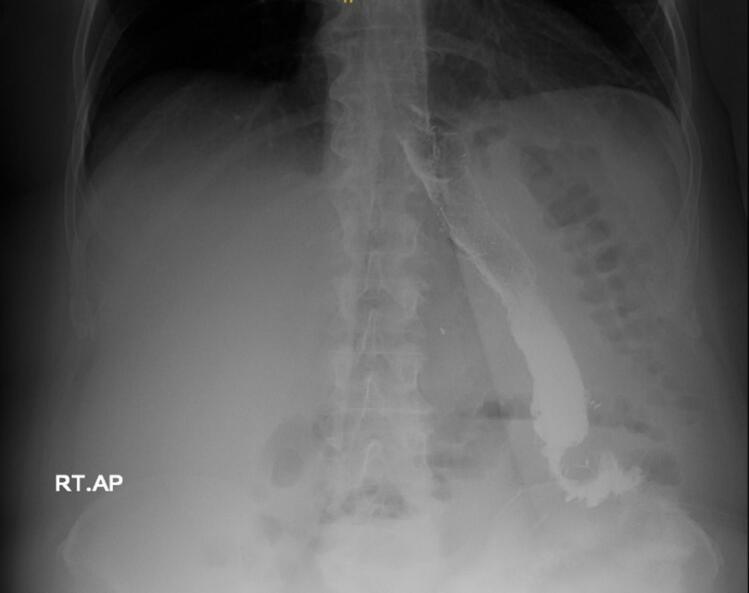
Contrast study following stent insertion.

The next day, an EGD with minimal inflation revealed a hyperemic wall cavity just distal to the gastroesophageal junction corresponding to the gastric pouch staple line. There was a questionable opening that corresponds to the location of GCF (Fig. 4). The GJ anastomosis was intact and healthy, with no evidence of marginal ulcers. An esophageal fully covered stent was placed, covering the disrupted staple line, and its position was confirmed with an X-ray (Fig. 5). Following stent insertion, oral intake was gradually started with a clear liquid diet with good tolerance and progressed to full liquids until she was able to meet 75 % of her nutritional needs. The patient was discharged with clear instructions and daily contact with her. After six weeks of outpatient follow-up, which was uneventful, endoscopic removal of stent was completed, and the patient was satisfied with the management plan.

## 3 Discussion

The rate of staple line disruption following RYGB is reported to be around 1 % [9]. The most common location for anastomotic disruption is GJ, with the gastric pouch being a relatively uncommon location. In the context of bariatric surgery, the cause of the anastomotic disruption is multifactorial. Some of these factors include the nature of the procedure (primary vs conversion/revision), patient's obesity related disease (diabetes mellitus, hyperlipidemia, hypertension), and habit related factors mainly smoking, and noncompliance to the postoperative plan (adequate fluid intake, discharge medications). Another factor that could be involved is oversewn the staple line which can lead to more ischemia and potentially anastomotic disruption. Consequently, early gastric fistula has the potential to develop [10]. Most anastomotic leaks will present with tachycardia and respiratory distress as early signs and symptoms [11]. Despite that, some patients can exhibit symptoms in an entirely atypical manner; our patient is a case in point.

The American Society of Metabolic and Bariatric Surgery endorses using endoscopic stents to manage anastomotic leak/fistula following RYGB [12]. There are reports in the literature demonstrating the success of managing early fistula after RYGB with endoscopic stenting, but none has reported a GCF managed with stent [[13], [14], [15], [16]]. With advancement of knowledge, several options for fistulas management has been developed, like clipping, tissue sealants, and double pigtube insertion. Yet stent insertion is still a great option for management of such cases [17,18]. GCF following RYGB is uncommon, and few case reports documented the management of this kind of complication mainly by surgical means, likely because all fistulae reported were late and already well formed (Table 1).

**Table 1:** Reported cases of GCF after gastric bypass.

Author	Type of procedure	Approach	Interval time (months)	Location	Management
Soo et al. [19]	Primary RYGB	NA	84	GJ	Laparoscopic resection and RYGB reversal
Gehle et al. [20]	Primary RYGB	Laparoscopy	10	GJ	Robotic resection and re-anastomosis
Musallam et al. [21]	Primary RYGB	NA	252	GJ	Conservative
Velleman et al. [22]	Primary RYGB	Laparoscopy	60	GJ	Failed primary repair. Resection and re-anastomosis

## 4 Conclusion

Our case is unique because of the unusual presentation, location of the leak, and type of fistula, as well as how it was managed non-surgically. Having a high level of suspicion in case of a patient's complaint is essential. Although surgical management should be considered in such presentation, nonsurgical management can be instituted with clinically stable patients. Adhering to the fundamentals in managing anastomotic leak/acute fistula, including stabilization, diagnostic imaging/endoscopy modalities, nutritional support, and expertise availability is crucial [12]. Patient counseling and education are crucial following such surgeries, which require a long-term commitment.

## Consent

Taken from the patient.

## Ethical approval

Exempted.

## Funding

None.

## Author contribution

Dr. Mohammad Almayouf: writing of paper, review of literature, collecting images.

Dr. Awadh Alqahtani: concept and overall review.

## Guarantor

Dr. Mohammad Almayouf.

## Research registration number

N/A.

## Conflict of interest statement

None.
